# An Uncommon Case of Disseminated Human Herpesvirus 6 Infection in an Immunocompetent Adult

**DOI:** 10.7759/cureus.102404

**Published:** 2026-01-27

**Authors:** Inês L Caetano, Joana V Cardoso, André S Silva, Eugénia Reiriz, Ana Cipriano

**Affiliations:** 1 Department of Infectious Diseases, Unidade Local de Saúde de Santo António, Porto, PRT

**Keywords:** exanthema subitum, hhv-6, human herpesvirus 6, immunocompetent adult, meningoencephalitis

## Abstract

Human herpesvirus 6 (HHV-6) is a common cause of infection in childhood, classically presenting as exanthema subitum. Viral reactivation causing severe disease in the setting of immunosuppression is well documented in the literature. However, HHV-6 infection in immunocompetent adults has been uncommonly reported.

We describe the case of an immunocompetent man in his 50s who presented with fever, headache, confusion, and a truncal maculopapular exanthema with sparse vesicular lesions. Lumbar puncture results were compatible with viral meningoencephalitis. HHV-6 DNA was identified in cerebrospinal fluid, serum, and vesicular skin lesions by polymerase chain reaction testing, supporting a diagnosis of disseminated HHV-6 infection with central nervous system and cutaneous involvement. The patient received treatment with ganciclovir followed by oral valganciclovir, with complete recovery and no sequelae.

This case highlights that HHV-6 may cause severe infection even in immunocompetent adults and underscores the need for evidence-based guidance to improve diagnosis and treatment of this condition.

## Introduction

Human herpesvirus 6 (HHV-6) belongs to the *Herpesviridae *family and includes two distinct species: HHV-6A and HHV-6B [1]. Given the ubiquitous nature of this microorganism, seroprevalence in the human population exceeds 90%, with the majority of infections occurring in early childhood and, when symptomatic, classically manifesting as exanthema subitum, also known as roseola infantum or sixth disease, which is predominantly caused by HHV-6B [1-3].

After primary infection, the virus establishes latency in specific cell populations, including peripheral blood mononuclear cells and central nervous system cells [4,5]. In the setting of immunosuppression, such as hematopoietic stem cell or solid organ transplantation or advanced human immunodeficiency virus infection, the possibility of HHV-6 reactivation is well established [1,2] and may be asymptomatic or present with a wide spectrum of clinical manifestations, ranging from mild disease with fever and rash to more severe conditions, particularly pneumonitis, hepatitis, colitis, and meningitis and/or encephalitis [2,6,7]. Additionally, HHV-6 is capable of integrating its genome into the telomeres of human chromosomes, with approximately 1% of the human population carrying the HHV-6 genome in all their nucleated cells, a condition termed chromosomally integrated HHV-6 (ciHHV-6) [2,4,8].

Diagnosis of HHV-6 infections relies primarily on polymerase chain reaction (PCR) assays. However, these methods have several limitations, including the inability to accurately differentiate between active infection, latent infection, and ciHHV-6 [2].

HHV-6 infection in immunocompetent adults, either primary infection or reactivation, is uncommon. Current literature on this condition is largely limited to case reports and small case series or narrative reviews [7-12], resulting in a lack of evidence-based guidance regarding its diagnostic and therapeutic approach.

This report describes a case of disseminated HHV-6 infection with viremia, cutaneous manifestations, and meningoencephalitis in an immunocompetent adult patient, with the aim of improving recognition and discussing the challenges in diagnosis and treatment of this clinical entity. 

This article was previously presented as an oral presentation at the 33rdEuropean Congress of Clinical Microbiology and Infectious Diseases on April 16, 2023.

## Case presentation

**Figure 1:**
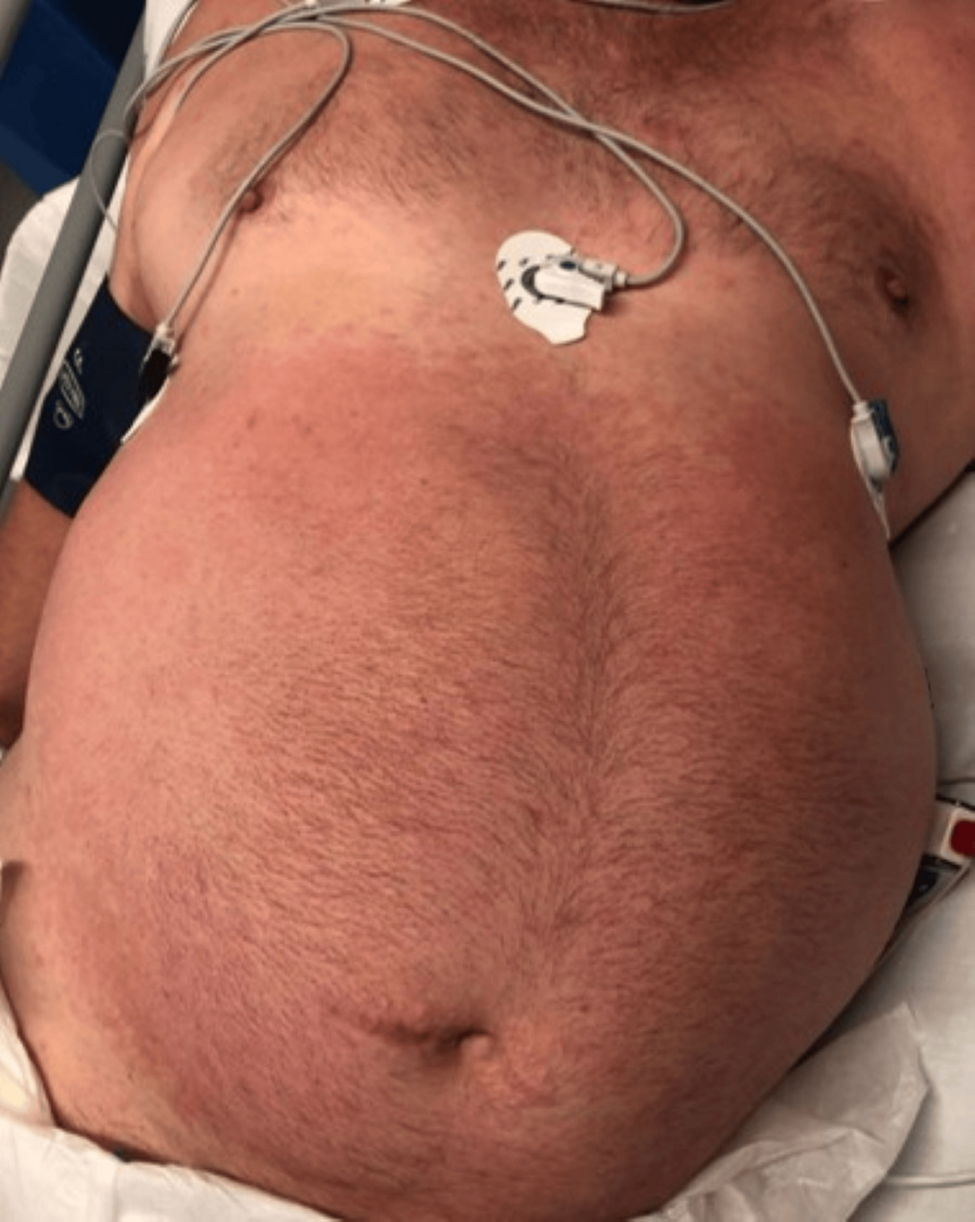
Maculopapular exanthema of the trunk on day 2 of hospitalization

We report the case of a male patient in his 50s with previous medical history of class 3 obesity, type 2 diabetes mellitus, hypertension, chronic alcohol consumption, severe obstructive sleep apnea, and a hospitalization six months prior for *Listeria monocytogenes *meningitis, having completed 21 days of ampicillin with a favorable outcome. He presented to the emergency department with a one-week history of headache, confusion, and fever. There was no relevant epidemiological context or recent exposure to antibiotics or new medications, although the patient had received a COVID-19 vaccine (Pfizer-BioNTech) three weeks prior to admission. On physical examination, the patient exhibited an altered mental status, with disorientation to time and place, confused speech, and inconsistent response to commands, as well as neck stiffness and a maculopapular exanthema on the trunk with sparse vesicular lesions (Figure 1). Arterial blood gas analysis demonstrated type 1 respiratory failure (PaO_2_/FiO_2_ ratio 193). Laboratory tests revealed only mild lymphopenia (1120 cells/µL), elevated lactate dehydrogenase (297 U/L) and slightly increased C-reactive protein (21 mg/L). Chest radiograph and head computed tomography (CT) scan showed no significant abnormalities. A lumbar puncture was performed, with cerebrospinal fluid (CSF) analysis identifying lymphocytic pleocytosis (56 leukocytes/µL, of which 54 were mononuclear cells), slight elevation of protein levels (0.88 g/L) and normal glucose, after which empirical treatment for meningoencephalitis with acyclovir, ampicillin and ceftriaxone was started.

On day two of hospitalization, the patient experienced worsening respiratory dysfunction, requiring transfer to the intensive care unit for high-flow nasal cannula (HFNC) oxygen therapy. In addition, significant labial and periocular edema developed, and the exanthema expanded to the limbs. CSF microbiological studies revealed positive PCR results for HHV-6 (quantification not available) and Epstein-Barr virus (below the quantification threshold), while PCR assays for other viral agents, namely cytomegalovirus (CMV), herpes simplex virus (HSV) types 1 and 2, and varicella-zoster virus (VZV) were negative; bacterial cultures were sterile. Possible HHV-6 infection with central nervous system and cutaneous involvement was considered the main hypothesis; therefore, intravenous ganciclovir 5 mg/kg twice daily was started, with discontinuation of the previously ongoing therapy. HHV-6 PCR testing in vesicular skin lesions and blood was subsequently performed, with positive results in both samples (quantification not available). Additional diagnostic investigations yielded the following results: serum Epstein-Barr virus PCR was positive, but below the quantification threshold; serological tests for *Rickettsia conorii*, *Borrelia burgdorferi*, HSV-1/2, VZV, CMV, and Epstein-Barr virus showed no evidence of acute infection (HHV-6 serology not available); negative serology for human immunodeficiency virus and syphilis; negative antineuronal and antinuclear antibodies; normal immunoglobulin and complement levels; brain magnetic resonance imaging and thoracoabdominopelvic CT scan revealed no clinically significant findings; lymphocyte immunophenotyping was compatible with active herpesvirus infection, demonstrating markers of cellular activation and redistribution of lymphocyte subsets in peripheral blood.

**Figure 2:**
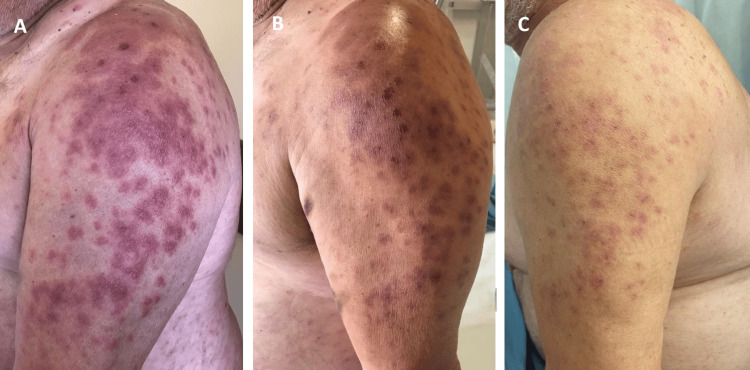
Evolution of cutaneous lesions on the upper limbs during hospitalization On days six (A), eight (B), and 19 (C) of admission

**Figure 3:**
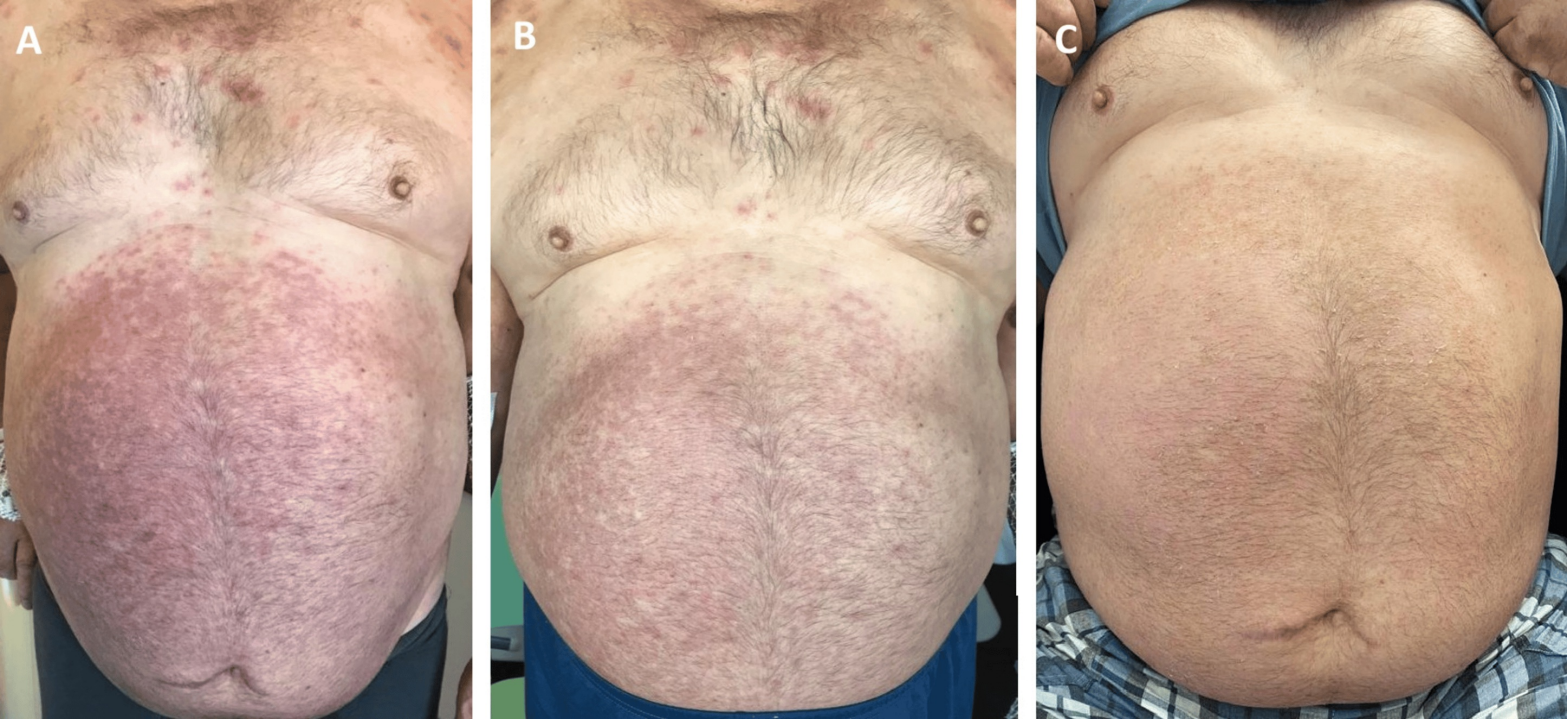
Evolution of cutaneous lesions on the trunk during hospitalization On days six (A), seven (B), and 19 (C) of admission

Following initiation of ganciclovir, the patient showed a favorable clinical course, with rapid resolution of neurological symptoms and respiratory dysfunction, alongside gradual improvement of the skin lesions (Figures 2, 3). After eight days of ganciclovir administration, treatment was transitioned to oral valganciclovir 900 mg twice daily, which was maintained until completion of a total of six weeks of therapy. The patient was discharged on day 19 of hospitalization and, at the three-month follow-up visit, he was asymptomatic, with no recurrence of neurological manifestations and complete resolution of the cutaneous findings.

## Discussion

Primary HHV-6 infection mostly occurs very early in life, while in adults, disease is mainly caused by viral reactivation, which is classically associated with immunosuppression [1,2]. Nonetheless, despite remaining uncommon, HHV-6 infection in immunocompetent individuals has been increasingly reported in the literature, particularly severe cases involving the central nervous system [7-11]. This condition represents a diagnostic challenge due to its variable and nonspecific clinical presentation, as well as limitations in currently available diagnostic assays [2].

HHV-6 central nervous system infection in immunocompetent adults generally presents with clinical and CSF features consistent with viral meningoencephalitis [5,12], similar to those observed in this case. Although cutaneous manifestations are well documented in primary HHV-6 infection during childhood [1], they appear to be infrequent and poorly characterized in immunocompetent adults; in our patient, their presence represented a notable finding that supported the diagnostic hypothesis. Neuroimaging studies may be normal or show variable findings, with limbic system involvement being the most typical [5,11]. A summary of previously reported cases, including clinical presentation, treatment, and outcomes, is provided in Table 1 for comparison with the present case.

**Table 1:** Summary of reported cases of HHV-6 central nervous system infection in immunocompetent adults, compared with the present case CSF: cerebrospinal fluid

Author (Year)	Type of Article	Clinical Presentation	HHV-6 PCR–Positive Samples	Treatment	Treatment Duration	Outcome
Yilmaz et al. (2018) [5]	Case report	Meningoencephalitis during pregnancy	CSF	Acyclovir	10 days	Clinical recovery
Alkozah et al. (2021) [10]	Case report	Meningitis, pneumonitis and viremia	CSF, serum, bronchoalveolar lavage	Ganciclovir followed by valganciclovir	3 weeks total (10 days intravenous followed by oral therapy)	Clinical recovery
Chia et al. (2024) [7]	Case report	Meningoencephalitis with viremia and hemophagocytic lymphohistiocytosis	CSF, serum	Ganciclovir followed by valganciclovir (+ corticosteroids)	54 days total (21 days intravenous followed by 33 days oral)	Clinical recovery
Webb et al. (2024) [8]	Narrative review (35 cases)	Encephalitis / meningoencephalitis	Mainly CSF	Ganciclovir, foscarnet, cidofovir, and acyclovir; valganciclovir used as oral step-down therapy in some cases	Inconsistently reported across cases	Heterogeneous; clinical recovery reported in many cases
Rodrigues Barbosa et al. (2025) [9]	Case report	Subacute gait ataxia, ocular symptoms and fever	CSF	Valganciclovir	10 days	Clinical recovery
Present case	Case report	Meningoencephalitis with rash and viremia	CSF, serum, skin lesions	Ganciclovir followed by valganciclovir	6 weeks total (8 days intravenous followed by 34 days oral)	Clinical recovery

HHV-6 PCR tests must be interpreted with caution, as positivity may reflect latent infection, active infection, or ciHHV-6. Quantitative assays are preferred over qualitative ones, since viral load may help distinguish between the different types of infection. Despite the absence of standardized thresholds for this categorization, persistently high HHV-6 DNA levels, usually exceeding one million copies per mL, are more consistent with ciHHV-6 than with active infection [1,2]. Additional diagnostic methods to evaluate the presence of ciHHV-6 include PCR testing of hair follicles or nail clippings, fluorescence in situ hybridization, and digital droplet PCR; however, these cannot be performed in many settings, restricting their clinical applicability [2,4,8].

In this case, viral load quantification and specific methods to detect ciHHV-6 were not available, which represents an important diagnostic limitation. Nevertheless, several factors favored active infection, including compatible clinical manifestations, multisite HHV-6 DNA detection, and favorable response to antiviral therapy, together with exclusion of alternative causes, such as other infections, allergic reactions, and autoimmune diseases. Concomitant low-level Epstein-Barr virus DNA detection below the quantification threshold did not explain the clinical presentation and was interpreted as an incidental finding. Drug reaction with eosinophilia and systemic symptoms, which may be associated with HHV-6 reactivation [13], was considered but deemed unlikely due to the absence of recent exposure to new medications and lack of eosinophilia.

Despite a prior episode of *L. monocytogenes* meningitis, no underlying cause of immunosuppression was identified in our case. Comorbidities such as obesity, diabetes mellitus, and chronic alcohol consumption have been linked to immune dysregulation [14,15], even though a direct association with HHV-6 reactivation has not been clearly established [16]. A temporal relation with COVID-19 vaccine administration was noted; however, HHV-6 reactivation in this setting appears to be rare and is not supported by robust clinical evidence [17]. 

Currently, there are no established treatment recommendations for HHV-6 infection in immunocompetent adults. Existing guidelines apply to transplant patients and reserve antiviral therapy for severe cases, particularly encephalitis, with ganciclovir, foscarnet, and cidofovir being considered the preferred agents [1,3,6]. In post-hematopoietic stem cell transplant patients, a treatment duration of at least three weeks, continued until viral clearance, is suggested [3]. A recent narrative review of cases of HHV-6 encephalitis in immunocompetent patients [8] demonstrated substantial variability in terms of the antiviral used, treatment duration, and oral step-down therapy, reflecting the lack of consensus on the optimal strategy, although at least 14 days of treatment at appropriate doses appeared to be sufficient to achieve clinical cure in most cases. In this setting, treatment decisions should be guided by disease severity and clinical response rather than standardized protocols.

## Conclusions

HHV-6 should be considered as a potential cause of meningoencephalitis and disseminated infection in immunocompetent adults, once alternative etiologies have been excluded. Cutaneous manifestations, which remain poorly characterized in this population, may provide an additional diagnostic clue. Interpretation of HHV-6 PCR results requires careful clinical, laboratory, and radiological correlation, especially in the absence of quantitative assays and specific testing for ciHHV-6. This report aims to raise awareness of this entity and support timely diagnosis and appropriate management in severe cases.
